# A Participatory Approach to Develop the Power Mobility Screening Tool and the Power Mobility Clinical Driving Assessment Tool

**DOI:** 10.1155/2014/541614

**Published:** 2014-09-08

**Authors:** Deepan C. Kamaraj, Brad E. Dicianno, Rory A. Cooper

**Affiliations:** ^1^VA Center of Excellence in Wheelchairs and Related Technology, VA Pittsburgh Healthcare System and Human Engineering Research Laboratories, Pittsburgh, PA 15206, USA; ^2^Human Engineering Research Laboratories, 6425 Penn Avenue, Bakery Square, Suite 400, Pittsburgh, PA 15206, USA; ^3^Department of Physical Medicine and Rehabilitation, University of Pittsburgh, Pittsburgh, PA, USA; ^4^Department of Bioengineering, University of Pittsburgh, Pittsburgh, PA, USA

## Abstract

The electric powered wheelchair (EPW) is an indispensable assistive device that increases participation among individuals with disabilities. However, due to lack of standardized assessment tools, developing evidence based training protocols for EPW users to improve driving skills has been a challenge. In this study, we adopt the principles of participatory research and employ qualitative methods to develop the Power Mobility Screening Tool (PMST) and Power Mobility Clinical Driving Assessment (PMCDA). Qualitative data from professional experts and expert EPW users who participated in a focus group and a discussion forum were used to establish content validity of the PMCDA and the PMST. These tools collectively could assess a user's current level of bodily function and their current EPW driving capacity. Further multicenter studies are necessary to evaluate the psychometric properties of these tests and develop EPW driving training protocols based on these assessment tools.

## 1. Introduction

Independent mobility is one of the most important determinants of quality of life for individuals with disabilities [1, 2]. Electric powered wheelchairs (EPWs) are key assistive technology devices that increase mobility and comfort while promoting social integration among users, thereby improving their overall quality of life [3, 4]. Over the past few decades, the number of EPW users has been increasing [5, 6] due to advances in health care, increase in the aging population of baby boomers, and the number of veterans returning from conflict situations [7–9]. However, this increase in EPW usage has also been associated with an increase in wheelchair related accidents, injuries [6, 10], and equipment abandonment [11, 12]. To compound this critical issue, it has also been shown that over forty percent of those who receive EPWs continue to have problems with certain EPW driving skills [13]. There also exists a growing cohort of people who desire and deserve EPWs for mobility but who have not been able to acquire a device because of severe impairments in motor, sensory, or cognitive functions that have precluded them from passing a clinical assessment or because of inadequate resources to allow them to practice driving [14–16].

Specific impairments in body structures and function, especially cognition and sensory perception, have been linked to problems with driving EPWs. Cullen and colleagues linked self-perceived functional performance of driving an EPW with verbal recall, visual construction ability, and global cognition [17]. Mendoza et al. reported anecdotal evidence that accidents among EPW users in a nursing home increased if the user had executive dysfunction [18]. Routhier et al. suggested that a variety of psychological factors influence EPW use: cognitive function, motivation, analytical capacity, and problem-solving [15]; and Batavia et al. noted that cognitive impairment affects EPW driving in users with traumatic brain injury [19]. Massengale et al. demonstrated that visual perception, visual function (specifically those with ocular motor function, stereo depth, field of vision, binocular vision, and far visual acuity), and cognition have a significant impact on EPW driving performance [20].

Currently, there are a few assessment tools available to rehabilitation professionals for evaluating EPW driving capacity, both in the user's natural environment and in the clinic. The Power Mobility Indoor Driving Assessment (PIDA) and the Power Mobility Community Driving Assessment (PCDA), developed by Dawson et al. and Letts et al., respectively, are the two most commonly used clinical tools that help identify general areas or tasks for which more training is needed (e.g., “parking under a table”) or where modifications to the EPW or the environment are necessary [14, 20, 21]. Following the PIDA and PCDA, Kirby et al. published the Wheelchair Skills Test (WST) that has mainly been used to evaluate manual wheelchair mobility [22] but more recently has been adapted for the evaluation of EPW driving skills as well [23]. Massengale et al. developed the Power Mobility Road Test (PMRT) to study the effect of visual function, visual perception, personality traits, and cognitive skills that affect EPW use [20]. The PMRT consists of a set of 12 structured tasks and 4 unstructured tasks with moving obstacles. The performance scores from these tasks were then correlated with outcomes from evaluations of visual function, visual perception, cognition, and personality traits to study their influence on EPW driving skills.

Apart from these widely used clinical tools, two other tools have been developed for research settings. The Functional Evaluation Rating Scale (FERS) developed by Hasdai et al. uses a scoring system similar to the PIDA and has been adopted by other researchers to assess driving performance in simulation programs [24, 25]. In a three-step process, Routheir et al. established a framework for wheelchair driving assessment [15], developed theObstacle Course Assessment of Wheelchair User Performance (OCWA UP) [26], and established reliability of the assessment tool [27].

However, even with advancements in technology to measure driving skill, there still are no standardized tools to screen potential adult EPW drivers for specific impairments in motor, sensory, or cognitive function [15–17, 20, 21]. Such a tool, coupled with a tool to measure driving skill, could lead to the development of clinical training protocols with the ultimate goal of improving independence and safety of potential drivers [16].

To better understand the concepts of function, activity, and capacity as they relate to EPW driving, Mortenson and colleagues evaluated wheelchair related outcome measures on the basis of the World Health Organization's (WHO) international classification of functioning, disability, and health (ICF) [28–31]. Mortenson et al. concluded that all of the currently available EPW driving assessment tools have been focused on evaluating wheeled mobility* capacity* or* performance* or both, to assess* activity and participation* of a wheelchair user [30]. However, none of the tools assess how the driver's physical and cognitive functions affect driving; in other words, it is also crucial to assess specific impairments in* body structures and function*.

There are multiple ways to approach this problem. Adopting previously established techniques used in adaptive vehicle driving is one such approach [32–36]. Driving rehabilitation specialists employ a series of tests that help identify impairments in major functional domains (motor or sensory or cognitive) that affect driving ability [33, 37–40]. If a driver has an impairment in one of the functional domains, targeted training programs in that specific domain and teaching compensatory mechanisms to overcome that impairment have been shown to improve driving performance [41–44]. Such an evidence based approach toward driving assessment and training has led to the development of sound clinical practice guidelines which have been effective in training and counseling drivers, such as the elderly [45].

Secondly, learning strategies and techniques that have been employed in training children with cognitive disabilities could provide valuable insight into the development of newer assessment and training tools for potential adult EPW users. Tefft et al. reported that problem solving and spatial relations had a direct impact on the variance of EPW driving skills among children [46]. Following Tefft et al., Furumasu et al. published the pediatric powered wheelchair skills test, which used a five-point scale to quantify a child's driving ability [47]. In 2011, Nilsson et al. reported several strategies that have been applied to teach EPW driving skills for children with cognitive disabilities [48, 49]. These studies have demonstrated the strong association of cognitive, sensory, and motor assessments with better training strategies in children [48].

In this study, we adopted the principles used in adaptive vehicle driving to screen for impairments and pooled them with the neuropsychiatric measures that have been used to measure capacity for EPW driving skills among adults, along with strategies and principles that have worked well with children to develop two new tools. Specifically, we employed participatory research [50–52] and qualitative methods [53, 54] to develop the Power Mobility Screening Tool (PMST) comprising a list of simple tests to quantify motor, sensory, and cognitive functioning (“pre-road” screening tool) and the Power Mobility Clinical Driving Assessment (PMCDA) (“on-road” test) to assess EPW driving capacity. The specific aim of this study was to establish content validity of both the PMST and the PMCDA.

## 2. Materials and Methods

### 2.1. Participants

This study was reviewed and approved by the University of Pittsburgh's Institutional Review Board. A convenience sample of 21 participants in the United States were approached by word of mouth or phone calls or via email to participate in the surveys and focus group phase of the study. The inclusion criteria were being a professional expert (physician, occupational therapist, physical therapist, or a rehabilitation engineer) in the field of assistive technology with at least five years of professional experience with the wheelchair delivery process or an expert EPW user who has been using an EPW for a minimum of three years, and the participant is between the ages of 18 and 80 years. There were no exclusion criteria.

A brief abstract explaining the purpose of the focus group and objective of the discussion forum was given to all the attendees as a part of the registration package of the 29th International Seating Symposium held in Nashville, TN, in 2013. Any attendee of the symposium who was interested in partaking was invited to participate in the discussion forum. There were no specific inclusion or exclusion criteria.

### 2.2. Research Protocol

#### 2.2.1. Surveys

Two separate surveys were sent to the professional experts and the EPW expert users via email: the Tools and Tasks survey (Appendix A) and the Users' survey (Appendix B). The purpose of these surveys was to generate a list of items that could be included in the PMST and the PMCDA, and rank these items based on the level of importance. The professional experts were asked to complete the Tools and Tasks survey, while the expert EPW users were asked to complete the User's survey.

The Tools and Tasks survey consisted of two sections. Section one was a list of tests commonly used to evaluate motor, sensory, and cognitive function in adaptive vehicle driving [33, 36, 40, 55], and section two consisted of a list of driver tasks pooled from existing EPW driving assessment tools [14, 20–22, 26, 56]. Participants were asked to rank each of the screening tests in order of importance within the motor, sensory, and cognitive sections. They were instructed to use ranks ranging from 1 (most important) to 3 (least important) (Appendix A). Similarly, for section two, a rank of 1 (most important) to 5 (least important) was requested (Appendix A). A rank of “0” was given if the test or task should not be included. The participants were also given an option to add more tests or tasks.

The User's survey consisted of two questions. Question one asked the participants to list the top 5 skills that are important for a person to be a highly skilled driver in both indoor and outdoor environments, and question two asked them to list the top 5 skills that are important for a person to be a moderately skilled driver who drives only indoors. The users were also asked to rank these tasks in the order of their importance within each question.

The surveys were sent to the participants two weeks before the scheduled date of the focus group and a follow-up reminder email alert to return all the surveys was sent one week before the focus group.

#### 2.2.2. Focus Group

After all the surveys were returned, a teleconference was set up for the focus group. Two researchers acted as moderators, and the entire focus group was audio recorded. The moderators presented the overall median rankings of the items and initiated a discussion using a structured set of questions [53, 54, 57] (Table 1). Following the focus group, the recording was transcribed and analyzed for common themes by each of the moderators individually. Then, the two moderators had a discussion to reach a consensus about predominant themes. Based on these themes and comments raised during the focus group, the first iteration of the PMST and PMCDA was established.

#### 2.2.3. Discussion Forum

Data from the surveys and the focus group were analyzed to develop the first iteration of the PMST and the PMCDA. Three months following the focus group, one of the moderators presented the first iteration of both tools in the discussion forum during the International Seating Symposium. A brief introduction of currently existing EPW driving tools was presented followed by the first iteration of the PMST and the PMCDA. The PMST and the PMCDA were further discussed, based on the structured set of questions listed in Table 1. Based on the comments put forth by the participants during the discussion forum, the second iteration of the PMST and PMCDA was developed (Tables 9 and 10).

## 3. Results

### 3.1. Surveys

Twenty-one experts were approached and invited to take the surveys, of which eight professional experts consented to participate. Of the ten expert EPW users approached, three consented to participate. All the eleven experts returned the surveys within two weeks (response rate of 100%). The mean duration of clinical experience of the professional experts was 13.75 (±6.9) years, and all of them had assistive technology professional (ATP) certifications. Tables 2 and 3 demonstrate the professional backgrounds of the eight experts who took the surveys and the 46 experts who participated in the discussion forum.  Table 4 shows the demographic profile of the expert EPW users. It is important to note that the only expert EPW user, who participated in the discussion forum, was also a rehabilitation scientist.

Table 5 shows successive iterations of the list of tests to be included in the PMST. Tables 6 and 7 show successive iterations of the list of tasks to be included in the PMCDA according to experts.  Table 8 shows the ranked list of tasks suggested by the users. Although the users were asked to list the top 5 skills, all the users listed more. During the focus group, one of the users pointed out, “These tasks are essential for an individual who receives his or her first EPW.”

### 3.2. Focus Group

All eight professional experts who took the surveys participated in the focus group. These experts defined essential criteria for the PMST and the PMCDA. The first criterion was that the tests should be easy to administer for therapists with any level of training (novice versus experienced) and with any professional background (occupation therapist versus physical therapist). As one of the physical therapists pointed out, “All physical therapists may not be trained to administer complex cognitive assessments… besides performing the mini mental status. So, we have to be clear that under my certification I can administer whatever test we choose to include, if this has to be a globally useful tool.” Secondly, the tests should be inexpensive and should not require the purchase of any supplies that are not commonly available in clinical settings. The same therapist also noted, “I do not have access to an accessible bathroom all the time. So, if we define a task like approaching or parking by a sink, I might not be able to administer it to all my clients… all the time.” Third, the scoring system should be clearly defined without any room for subjectivity. Experts agreed that a common problem with currently available tests is that the scoring systems are too complicated or subjective. One of the therapists indicated, “Either the 1 to 4 or 0 to 100 might provide a good system, but if it's not clearly defined, then the room for subjectivity is where it gets challenging.” These criteria led to the common consensus that the list of five screening tests (Table 5) would be sufficient to assess a user's functional capacity to drive an EPW. Similarly, the list of ten indoor tasks (Table 6) and ten outdoor tasks (Table 7) should not only be sufficient to assess users' safety and EPW driving capacity but would also help therapists identify clearly what area would require more user training.

Analysis of the transcripts of the focus group led to the identification of important thematic concepts for the tools. The group suggested that separate sections are essential for assessing driving capacity in the indoor and outdoor environments, as driving under these two circumstances has different skill sets. Hence, it was recommended that the PMCDA be designed to have two sections with tasks ordered by increasing level of complexity. The group agreed that the number of tasks in the PMCDA is sufficient to assess the baseline driving capacity and safety of the EPW user. Further, they felt most testers would require few supplies to conduct testing with either of the tools. The experts ranked eight tasks as “0,” indicating these tasks could be excluded from the assessment tool. However, during the development of the first iteration of the PMCDA, two of these tasks from the indoor section (drives backward or reverse 10 ft in a straight line and turns 90° while moving backward) and two of these tasks from the outdoor section (ascends 10° incline and descends 10° incline) were added to the list, since the users had ranked these skills highly and had indicated that these are essential skills necessary for a new EPW user.

Several themes emerged regarding the scoring system. The experts felt that the possible total scores on both tools must have a wide range in order to stratify drivers with variable functional capability and driving capacity. For example, a dichotomous pass or fail system should be avoided, since this system might not provide the sufficient variations in scores to include drivers with all skill levels. The possible total scores of 5–15 on the PMST and 23–69 on the PMCDA were felt to be sufficient for stratification. They also felt strongly that the scoring used for individual tasks should be clear and mutually exclusive and suggested that a score of “0” on the tools should be avoided. Based on these concerns, a three-point scoring system was proposed for individual tasks within both the PMST and PMCDA (Appendix C).

### 3.3. Discussion Forum

Among the 1300 attendees of ISS, 46 therapists, durable medical equipment suppliers, and rehabilitation technicians and one wheelchair user with cerebral palsy participated in the discussion forum. The discussion forum followed the same protocol as the focus group. Several salient issues were identified following the analysis of the transcription from the audio-recorded discussion forum. Overall, the group confirmed that all the tasks listed in the PMCDA are essential for the assessment of EPW driving capacity. In addition to the tasks listed in the first iteration, three other tasks were suggested for the indoor section of the PMCDA. First, “turning 90° and entering a doorway” was added since the group suggested this task is essential for safe driving and is a frequent occurrence in the user's natural environment. Secondly, “stopping the chair on command” was added, since participants proposed that this task was not only an assessment of the user's EPW driving capacity, but also a gauge of the EPW users' ability to respond to dynamic changes in their environment, which in turn reflects their ability to use the EPW safely. Lastly, “parking an EPW parallel to a transfer surface,” which could either be a bed or a chair, was also added, since participants felt this is a vital task that every EPW user will have to perform at some point in time irrespective of his or her medical need for using an EPW. It was suggested that this last task could be performed during the mat assessment typically performed during a routine examination for an EPW [58].

One task that was discussed extensively during the discussion forum was the ability of an EPW user “to get on and off an elevator.” Users also indicated this as one of the tasks that should be performed by an EPW user with moderate skill. However, this task was not added to the list for two reasons. First, it may not be feasible to administer the task in all clinics. And secondly, experts felt that two other tasks included in the list, namely, “can safely maneuver in-between 2 chairs spaced 32 inches apart” and “turns 180° in place to the left/right,” assess basic skills also necessary for elevator use. However, the group agreed that, during training, the trainer should make this an essential task to be practiced and discussed with the driver.

The group noted that objective measures to quantify users' functional impairments were essential, and performing functional clinical tests rather than lengthy neuropsychiatric or motor measures was advisable and acceptable. However, the group suggested two changes in the PMST. First, under the sensory section, the group pointed out that the term “visually” may not be applicable to all EPW users and will have to be changed to accommodate individuals with all levels of sensory functioning. Second, under the cognitive assessment, it was suggested that estimated length of time should be changed to “the entire period of assessment.” Finally, a change in the scoring system was also suggested. The criterion definition of scoring level 2 should be changed to include all kinds of cues (visual, verbal, and auditory) that encompass users with any functional capabilities. Based on these comments and suggestions, the second iteration of the PMCDA and the PMST was developed (Appendix C).

## 4. Discussion

Over the years, there has been a growing need for testing and developing wheelchair-specific outcome measures that allow clinicians to justify their equipment recommendations and to show the effectiveness of specific interventions to the scientific community [30]. By adopting the principles of ICF, such outcome measures can delineate and define methods to assess body structures and function, tasks important for activities and participation [30], and the capacity and performance of the user [28, 30, 59]. Design of outcome measures for EPW driving should also follow few key principles that have been recognized as salient in the scientific literature. First, the goal of the assessment should be explicitly targeted towards enhancing mobility and independence of the user rather than preventing access to EPWs for potentially unsafe drivers [21]. In other words, the measure should be used with the goal of assessing safety and identifying areas where training can help a potential driver improve skills, rather than simply determining whether he or she is capable of driving at one point in time. Second, the measure should be scored in such a way that it can demonstrate progress with training [15, 26, 27]. Third, the measure should be able to identify key areas where training could improve skill [20], not only by identifying what tasks are difficult for a driver but also by identifying what body structures and functions are contributing to those specific difficult tasks. The experts and users in this study reinforced these principles, and the participatory approach that was adopted accommodated all three principles when developing the tools. The iterative approach, with inclusion of over 50 professional experts and expert EPW users, established good content validity for the PMST and the PMCDA.

Although we adopted concepts from adaptive vehicle driving literature to develop the content of the PMST, the tool that emerged is uniquely suited for EPW driving. Experts identified several concerns in administering many of the standardized neuropsychological tests commonly used for vehicle driving in a wheelchair clinic. First, the qualifications and training necessary to administer these tests might preclude use by many potential testers. Second, each of the tests would require the clinic to purchase a test kit, and if multiple tests were to be administered together, it would result in an expensive assessment process. Third, the process would become quite lengthy, which decreases the likelihood of a tester offering these tests in a busy wheelchair clinic. Most importantly, they excluded many of the tests because they did not feel that the tools were sensitive or specific enough to measure functional capability in terms of EPW driving ability. However, the experts did agree that objective measures are necessary in each of the three domains (motor, sensory, and cognitive) to quantify a user's functional impairments in these domains. Hence, rather than using standardized neuropsychological tests, the experts proposed the use of functional clinical tests (Table 4) for the purpose of screening. If any major clinical concerns would be identified during screening, then the experts recommended use of the PMST as a basis for referral for further testing by a specialist such as a neuropsychologist, an audiologist, or an ophthalmologist.

The content of the PMCDA includes similar driving tasks as those identified by a focus group conducted by Torkia et al. [60]. In that study, researchers identified four specific wheelchair mobility tasks/maneuvers that were difficult for EPW users, namely, controlling the EPW's joystick, avoiding obstacles, maneuvering backwards, and going through narrow doorways. In addition, this study also reported that, during outdoor mobility, EPW users face difficulty in four major areas: using streets and sidewalks, navigating through crowds, using adapted modes of transportation, and dealing with rain or snow conditions. Although our tool does not include measures of transportation or inclement weather for practical reasons, it is worthwhile to note the striking similarities in the other tasks identified in their study.

There are significant advantages to using the PMST and the PMCDA in combination as a tool kit to assess EPW driving capacity over the currently existing tools. Currently, there are no other validated methods of* screening* for cognitive, motor, and sensory issues related to EPW driving. This is the first time a screening tool with functional tasks has been validated dually with an assessment for EPW driving in adults. A validated screening tool for cognitive, motor, and sensory capacity may help to standardize the evaluation process if adopted across centers. This, in turn, could lead to the development of training interventions customized for each type of impairment that affects various driving skills. Development of such customized interventions is important because individuals with cognitive or sensory impairments may need extra training and should not be excluded from opportunities to learn to drive based on a sole screening or assessment. Rather, this combination of tools can help to identify areas that would need more training to make the user a better EPW driver. Another advantage to using the PMST and the PMCDA is that they are pure measures of driving capacity; that is, they include only tasks that are exclusively related to EPW driving, not other factors like wheelchair maintenance. Lastly, as reported by one of the participants during the discussion forum, because they have a clearly defined scoring system, the PMST/PMCDA combination tool kit is quick and easy to administer without ambiguity among scoring levels.

### 4.1. Study Limitations

Because the experts were identified through a convenience sample of colleagues and acquaintances in the field of assistive technology, they may have been following similar clinical practices as the investigative team, which may have made it easier to reach consensus on content validity. However, participants were recruited from several locations across the country and inclusion of a large number of participants from the discussion forum who were voluntarily attending the session increased the diversity of the input. Still, the tool was developed solely using input from American and Canadian experts and is not validated for other cultures or languages. Offering the survey only via email limited the external validity because not all EPW users necessarily have computers. However, using email also provided the ability for some users to participate who might not otherwise been able to participate due to transportation barriers. The large number of participants in the discussion forum could have hindered some participants from expressing their views. However, we allowed ample time for individual questions and comments after the discussion forum ended, which provided the moderator with an opportunity to incorporate individual questions and concerns in the iterative revision of tools. In addition, both the professional experts and the expert users were included in one group for the focus group and the discussion forum. One benefit of having this structure was that participants were able to hear opinions that may be quite different from their own. On the other hand, diversity within a focus group can sometimes cause the group to stray from the topic or have trouble honing in on specific ideas. However, the latter was not a problem in this study as the group was closely moderated and sufficient content was produced to be useful for tool development. Finally, this study included only four expert EPW users, in comparison to the fifty professional experts in the study. However, tasks pooled from the past literature combined with the Users' survey were helpful in identifying key tasks for the PMCDA, which have also been identified by users in another focus group study [60].

### 4.2. Future Directions

Further testing is needed to evaluate feasibility of administering this tool in a busy clinical environment, and further psychometric testing is needed to establish inter- and intrarater reliability. The development of a strong clinical tool is an iterative process and, hence, future work will include a wider range of users with varying degrees of sensory or cognitive disabilities. In the future, we will develop a normative dataset that displays the functional capabilities of a wide range of EPW users, which would then help us develop training protocols targeted towards specific motor, sensory, or cognitive impairments that affect driving skill which are identified by the PMST. We intend to work with expert EPW users to identify strategies and techniques that could help teach newer users and marginally skilled users to drive better, based on their functional ability. Finally, we wish to study the effectiveness of such a training intervention on driving outcomes.

## 5. Conclusion

The scientific literature is sparse in measurements that can quantify a spectrum of driving skills among adult EPW users, and prior to this study, no screening tool for motor, sensory, and cognitive impairments that could impact EPW driving existed. This study used a participatory approach to establish content validity of a new screening tool for these impairments and an assessment tool to quantify EPW driving performance. Further work is necessary to establish the feasibility and reliability of these assessment instruments and to build and test training protocols for EPW driving. Multisite testing in large populations of EPW users is needed.

## Figures and Tables

**Table 1:** Questions for the focus group.

Screening tool	
What sections should it contain?	
What tests should be included under each section and how many?	
Can the tests be used in people with high-level motor impairment?	
How should this tool be scored?	
How long will it take to complete?	
What supplies are needed?	

Assessment tool	
What sections should the tool have?	
What tasks should be in each section?	
How should it be scored?	
How should each task be defined or delineated?	
How long will it take to complete?	
What supplies are needed?	

**Table 2:** Professional background of the professional experts who took the surveys and participated in the focus group.

Professional background	Number of participants	Mean years of experience (years ± SD)	Min (years)	Max (years)
Physical therapists	4	13.75 (9.39)	5	26
Occupational therapists	3	14.66 (5.68)	10	21
Rehabilitation scientist	1	11	—	—

Total	8	13.75 (6.94)	5	26

**Table:** (a) Professional background

Professional background	Number of participants
Physical therapist	20 (44%)
Occupational therapists	10 (22%)
AT supplier	14 (31%)
Others (rehabilitation technicians and rehabilitation engineers)	2 (3%)

Total	46 (100%)

**Table:** (b) Years of experience

Years of experience	Number of participants
0–2 years	1 (2%)
3–5 years	0
6–10 years	12 (26%)
More than 10 years	12 (26%)
More than 20 years	13 (28%)
More than 30 years	7 (16%)
More than 40 years	1 (2%)

**Table 4:** Medical diagnosis and years of EPW usage of the users who participated in the study.

	Medical diagnosis	Number of years of EPW usage
Surveys and focus group	Cerebral palsy	17
	SCI	5
	Connective tissue disorders with multiple orthopedic abnormalities	4.5

Discussion forum	Cerebral palsy	21

**Table 5:** Successive iterations of the screening tests.

Ranked screening tests (from the survey)		List of screening tests in the first iteration of the PMCDA (after the focus group)	Specific changes suggested during the discussion forum
Professional experts' ranks	Screening tests		
1	(i) Range of motion of the head, neck, and trunk [61, 62] (ii) Others: (a) Knowledge of cause and effect (b) Motor planning/problem solving ability, for example, maneuvering out of a tight spot (c) Basic cognition: orientation to person, place, or situation	Motor (i) Driver can functionally control an interface (joystick, head control, etc.) with appropriate body part to drive the chair (ii) Driver controls chair with sufficient endurance (ability to tolerate sitting and operating the control interface) during the screening Sensory (i) Driver can **visually identify** an object (e.g., therapy ball) 2 meters away with clinic in background, in left, center, and right visual fields Cognitive (i) Driver displays ability to understand cause and effect (action on the control interface will move the chair) (ii) Driver has ability to focus, concentrate, attend to task, and shift focus within the task	Sensory (i) Driver can **identify **an object (e.g., therapy ball) 2 meters away with clinic in background, in left, center, and right visual fields Cognitive (i) Driver has ability to focus, concentrate, attend to task, and shift focus within the task **during the entire period of assessment**
1.5	(i) Confrontation testing [63, 64] (ii) Snellen's chart (for far vision) [65] (iii) Random Dot Stereoacuity test [66] (iv) Others: (a) Strength of the body part that will be controlling chair (b) Ability to use control interface, for example, switch, joystick, and so forth		
2	(i) Range of motion of the upper limbs [67, 68] (ii) Near vision acuity charts [69] (iii) Proteus maze [70] (iv) Continuous performance test [71] (v) Others: (a) Minimental exam		
2.5	(i) The NSUCO/Maples oculomotor test [72] (ii) Motor-free visual perception test (MVPT) [73] (iii) Digit span [74, 75]		
3	(i) Motor coordination [76, 77] (ii) Trail making A & B [78] (iii) Others: (a) Functional vision—visual scanning, visual conflict (b) Endurance with use of trial equipment with driving obstacles (c) Reliability of use of the control interface (nonfatigable, consistent)		

**Table 6:** List of indoor driver tasks.

Ranked indoor driver tasks from the survey		List of indoor tasks in the first iteration of the PMCDA after the focus group	Specific changes suggested during the discussion forum
Professional experts' ranks	Indoor driver tasks		
1	(i) Drives forward (15 ft) (in a straight line) in narrow corridor without hitting walls (ii) Avoids one person coming towards participant in hallway	(i) Drives forward (15 ft) (in a straight line) in 36′′ hallway (ii) Drives backward 10 ft in a straight line in 36′′ hallway (iii) Passes through 36′′ doorway (iv) Avoids therapy balls approaching from left and right (v) Turns 90° while moving forward (vi) Turns 90° while moving backward (vii) Turns 180° in place to the left (viii) Can safely maneuver in-between 2 chairs spaced 32 in apart (ix) Approaches an accessible sink (x) Negotiates over a 1 in door threshold or mock threshold (piece of wood)	These tasks were added to the list: (i) Turns 90° and enters a doorway (ii) Approaches a transfer surface (bed or chair) (iii) Stops on command (emergency stop)
2	(i) Turns 90° while moving forward (ii) Passes through doorways without hitting walls (36′′doorways)		
3	Turns 180° in place—left		
4	Can safely maneuver in-between objects and tight spaces		
5	Approaches furniture without bumping into them		
0	(i) Drives backward (or reverse) 10 ft, in a straight line (ii) Turns 90° while moving backward (iii) Avoids “wet floor” sign (within a 5 ft wide corridor) (iv) Parking under table (v) Parking beside table		

**Table 7:** List of outdoor driver tasks.

Ranked outdoor driver tasks from the survey		List of outdoor tasks in the first iteration of the PMCDA after the focus group	Specific changes suggested during the discussion forum
Professional experts' ranks	Outdoor driver tasks		
1	(i) Avoids moving obstacles approaching from both sides—left and right (ii) Drives forward 30 ft in 30 s	(i) Drives forward 30 ft in 30 s (ii) Drives over an unpaved surface (iii) Ascends 5° incline (iv) Descends 5° incline (v) Ascends 10° incline (vi) Descends 10° incline (vii) Crosses a street (viii) Rolls 10 ft across 5° side-slope (ix) Ascends an ADA^1^ curb cut (x) Descends an ADA^1^ curb cut	No additional tasks suggested
2	Ascends 5° incline		
3	Descends 5° incline		
4	(i) Crossing street without lights (ii) Rolls 10 ft across 5° side-slope		
0	(i) Ascends 10° incline (ii) Descends 10° incline (iii) Is able to drive over 15 cm pot-hole		

^1^American Disabilities Act.

**Table 8:** Essential EPW driving tasks suggested by expert EPW users.

*Indoor skills *
(1) Carrying out skills in reverse direction (doorways, navigating around objects)
(a) Driving backwards in various environments
(2) Navigating around objects (couches, chairs, tables)
(a) Knowing where people are
(b) Knowing where tables and chairs are
(c) Navigating in crowded environments
(3) Negotiating tight doorways
(a) Navigating in narrow hallways and doorways (just wide enough for chair)
(4) Turning around in tight places (elevators)
(5) Parking next to transfer stations (bed, toilet)
(6) Speed control
(7) Paying attention to corners
(8) Paying attention to areas with stairs
(9) Opening and entering a door with an auto-closer
(10) Navigating over wet tile (hydroplaning)

*Outdoor skills *
(1) Looking everywhere before moving
(2) Always staying to one side of the hallway or sidewalk
(3) Combinations of skills (starting and stopping on ramps, etc.)
(a) Driving up and down steep grades
(b) Boarding public buses
(4) Turning around on cross slopes
(a) Driving straight on cross slopes
(5) Navigating ramps
(6) Driving on rough terrain (broken sidewalks, gravel, brick)
(a) Paying attention to bumps and edges
(b) Maintaining desired driving route on uneven ground such as cobblestone, brick, and gravel
(c) Climbing over obstacles (uneven curb cuts, sidewalks, small curbs)
(7) Starting and stopping quickly
(a) Speed control
(b) Ability to stop fast

*Indoor skills for moderate drivers *
(1) Navigating around objects
(2) Negotiating doorways
(a) Driving in ADA accessible hallways and doorways
(b) Opening, passing through, and closing a door
(c) Entering and exiting elevator and turning around inside as necessary
(3) Parking next to a transfer area (bed, toilet, mat table)
(a) Pulling under or up to side of table/counter
(b) Parking in desired space for transfer
(4) Turning around in open space
(5) Object avoidance
(a) Speed control
(b) Paying attention to corners
(c) Paying attention to areas with stairs
(d) Knowing where people are
(e) Knowing where tables and chairs are

**Table 9:** The power mobility screening tool (PMST).

Motor	
Driver can functionally control an interface (joystick, head control, etc.) with appropriate body part to drive the chair	1–3
Driver controls chair with sufficient endurance (ability to tolerate sitting and operating the interface)	1–3
Sensory	
Driver can identify an object (e.g., therapy ball) 2 meters away with clinic in background, in left, center, and right visual fields	1–3
Cognitive	
Driver displays ability to understand cause and effect (action on the control interface will move the chair)	1–3
Driver has ability to focus, concentrate, attend to task, and shift focus within the task during screening	1–3

Total	5–15

See Appendix C.1 for instructions on using the PMST.

**Table 10:** The power mobility clinical driving assessment tool (PMCDA).

	Score
Indoor	
Drives forward (15 ft) (in a straight line) in 36′′ hallway	1–3
Drives backward 10 ft in a straight line in 36′′ hallway	1–3
Passes through 36′′ doorway	1–3
Avoids therapy balls approaching from left and right	1–3
Turns 90° while moving forward	1–3
Turns 90° and enters a doorway	1–3
Turns 90° while moving backward	1–3
Turns 180° in place to the left	1–3
Can safely maneuver in-between 2 chairs 32 in apart	1–3
Approaches an accessible sink	1–3
Approaches a transfer surface (bed or chair)	1–3
Negotiates over 1 in door/mock threshold (piece of wood)	1–3
Stops on command (emergency stop)	1–3

Outdoor	
Drives forward 30 ft in 30 s	1–3
Drives over an unpaved surface	1–3
Ascends 5° incline	1–3
Descends 5° incline	1–3
Ascends 10° incline	1–3
Descends 10° incline	1–3
Crosses a street	1–3
Rolls 10 ft across 5° side-slope	1–3
Ascends an ADA curb cut	1–3
Descends an ADA curb cut	1–3

Total	23–69

See Appendix C.2 for instructions on using the PMCDA.

## Appendices

### A. The Tools and Tasks Survey

#### A.1. Screening Tools

Please choose 3 items in each section that are the most important components of a screening for power mobility. Rank them in order from 1 (most important) to 3 (least important) and leave the rest blank. You may choose a combination of existing items or write in your own, but please list only a total of 3 items in each section.

##### A.1.1. Motor: Choose 3 Here

1. Range of motion of the upper limbs
2. Range of motion of the head, neck, and trunk
3. Motor coordination
  - Purdue pegboard: measures two types of activities: gross movements of hands, fingers, and arms and “fingertip” dexterity in an assembly task. Involves sequential insertion of pegs and assembly of pegs, collars, and washers
  - Grooved peg board/fine motor speed-manipulative dexterity test using holes with randomly positioned slots and pegs, which have a key along one side. Pegs must be rotated to match the hole before they can be inserted.
4. Others: …

##### A.1.2. Sensory: Choose 3 Here

1. Visual
  - * Ocular movement*: the* NSUCO/Maples oculomotor test* is a standardized method of scoring standard eye movement testing.
  - * Visual field* (*by confrontation testing*)
  - * Visual acuity*
    - * Snellen's chart* (for far vision)
    - * Near vision acuity charts*: charts that can assess vision within 1 m distance.
  - * Depth perception (stereopsis)*:* Random Dot Stereoacuity test: *designed to rapidly test for amblyopia and strabismus in early and nonreaders and nonverbal children and adults.
  - * Color vision*:* color vision testing made easy*: intended use is for screening color vision of young children beginning at age 3 and individuals with developmental delays.
  - * Visual perception*
    - * Motor-free visual perception test (MVPT-3)*: assesses an individual's visual perceptual ability—with no motor involvement needed to make a response
    - * Developmental test of visual perception adolescent and adult (DTVP-A)*: a comprehensive measure of visual perception that reliably differentiates visual-perceptual problems from visual-motor integration deficits.
  - Others: …
2. Auditory
  - * Calibrated finger rub auditory screening test (CALFRAST)*: confrontational testing using fingers to make audible sound
  - * Portable audiometer*
  - Others: …

##### A.1.3. Cognitive: Choose 3 Here

1. Cognition and memory skills
  - * Trail making A *&* B*: specifically assesses working memory, visual processing, visuospatial skills, selective and divided attention, and psychomotor coordination.
  - * Clock drawing test*: assesses a patient's long-term memory, short-term memory, visual perception, visuospatial skills, selective attention, abstract thinking, and executive skills. Preliminary research indicates an association between specific scoring elements of the clock drawing test and poor driving performance.
2. Porteus maze: set of paper forms on which the subject is required to trace a path; tests problem solving
3. Digit span (WSIR): tests speed of information processing, longest list of letters or numbers that a person can repeat back in correct order
4. Continuous performance test by Connors (CPT): tests visual attention; task-oriented computerized assessment of attention disorders. Clients are presented with a repetitive, “boring,” task and must maintain their focus
5. Others: …

#### A.2. Driving Tasks

Please choose 5 items in each section that are the most important components of a driving skills assessment. Rank them in order from 1 (most important) to 5 (least important) and leave the rest blank. You may choose a combination of existing items or write in your own, but please list only a total of 5 items in each section.

*Indoor*

1. Drives forward (15 ft) (in a straight line) in narrow corridor without hitting walls
2. Drives backward (or reverse) 10 ft, in a straight line
3. Turns 90° while moving forward
  - Left
  - Right
4. Turns 90° while moving backward
  - Left
  - Right
5. Turns 180° in place
  - Left
  - Right
6. Passes through doorways without hitting walls (36′′ doorways)
7. Avoids “wet floor” sign (within a 5 ft wide corridor)
8. Avoids one person coming towards participant in hallway
9. Can safely maneuver in-between objects and tight spaces
  - Drive between a couch and coffee table, in a living room setup
  - Can enter an elevator
  - Adjust within an elevator
  - Exit the elevator
10. Approaches furniture without bumping into them
  - Parking under table
  - Parking beside table

Others:

(11) …
(12) …
(13) …
(14) …
(15) …

*Outdoor*

1. Drives forward 30 ft in 30 s
2. Crossing street without lights
3. Avoids moving obstacles approaching from both sides: left and right
  - Avoids two or more moving obstacles (person coming towards participant) in sidewalk
  - Avoid an unexpected ball
4. Ascends 5° incline
5. Descends 5° incline
6. Ascends 10° incline
7. Descends 10° incline
8. Rolls 10 ft across 5° side-slope
  - Left
  - Right
9. Is able to drive over 15 cm pot-hole

Others:

(10) …
(11) …
(12) …
(13) …
(14) …

### B. Users' Survey

*Years of Experience Driving a Power Chair* …

*Main Disability* …

*Question 1.* List the top 5 skills that you think are important for a person to be* a highly skilled driver in both indoor and outdoor environments*. Please rank them in order of importance from most important to least important.

*Indoor Skills*

1. * *
2. * *
3. * *
4. * *
5. * *

*Outdoor Skills*

1. * *
2. * *
3. * *
4. * *
5. * *

*Question 2.* List the top 5 skills that you think are important for a person to be* a moderately skilled driver who drives only indoors*. Please rank them in order of importance from most important to least important.

*Indoor Skills*

1. * *
2. * *
3. * *
4. * *
5. * *

Please include any additional comments below.

### C. Instructions for Using PMST & PMCDA

#### C.1. The Power Mobility Screening Tool (PMST)

See Table 9.

*Instructions*

- Ask client to drive the EPW in an open space free from obstacles.
- You may provide visual or auditory clues along with verbal instructions to complete tasks.
- Tasks can be completed in any order and also as part of a routine physical examination or mat assessment.
- Client may identify objects by any means (verbally, gestures, etc.) and may use visual aids.
- Control interface settings should be adjusted for safety and at discretion of the trainer and driver.

*Scoring System for the Screening Tool*

Score of 1: if the driver requires physical assistance, lacks the skill, or cannot complete the task, a score of 1 is given.
Score of 2: if the driver requires verbal or auditory hints or cues but no physical assistance or has partial skill (e.g., can identify an object in 2 of 3 visual fields or can partially move a joystick) a score of 2 is given.
Score of 3: if the driver completes the task without help or has adequate skill, then a score of 3 is given, even if additional time is needed for the task.

#### C.2. The Power Mobility Clinical Driving Assessment Tool (PMCDA)

See Table 10.

*Instructions*

- You may provide visual or auditory clues along with verbal instructions to complete tasks.
- Tasks can be completed in any order.
- Control interface settings should be adjusted for safety and at discretion of the trainer and driver.

*Scoring System for the Driving Assessment Tool*

Score of 1: if the driver requires physical assistance or cannot complete the task, a score of 1 is given.
Score of 2: if the driver requires verbal or auditory hints or cues but no physical assistance, a score of 2 is given.
Score of 3: if the driver completes the task without help, then a score of 3 is given.

## Abbreviations

EPW: Electric powered wheelchair
ICF: International Classification of Functioning, Disability, and Health
WHO: World Health Organization
PMST: Powered Mobility Screening Tool
PMCDA: Powered Mobility Clinical Driving Assessment Tool
PIDA: Power Mobility Indoor Driving Assessment
PCDA: Power Mobility Community Driving Assessment
WST: Wheelchair Skills Test
PMRT: Power Mobility Road Test
FERS: Functional Evaluation Rating Scale
OCAW UP: Obstacle Course Assessment of Wheelchair User Performance
ADA: American Disabilities Act.
